# Synthesis, Antiproliferative, and Antioxidant Evaluation of 2-Pentylquinazolin-4(3*H*)-one(thione) Derivatives with DFT Study

**DOI:** 10.3390/molecules24203787

**Published:** 2019-10-21

**Authors:** Amira A. El-Sayed, Mahmoud F. Ismail, Abd El-Galil E. Amr, Ahmed M. Naglah

**Affiliations:** 1 Department of Chemistry, Faculty of Science, Ain Shams University, 11566 Abbassia, Cairo 11566, Egypt; amira_aa47@hotmail.com (A.A.E.-S.); fawzy2010@sci.asu.edu.eg (M.F.I.); 2 Pharmaceutical Chemistry Department, Drug Exploration & Development Chair (DEDC), College of Pharmacy, King Saud University, Riyadh 11451, Saudi Arabia; amnaglah@gmail.com; 3 Applied Organic Chemistry Department, National Research Center, Cairo, Dokki 12622, Egypt; 4 Peptide Chemistry Department, Chemical Industries Research Division, National Research Centre, Dokki, Cairo 12622, Egypt

**Keywords:** quinazolin-4(3*H*)-one, quinazolin-4(3*H*)-thione, Schiff base, antiproliferative activity, antioxidant activity, DFT study

## Abstract

The current study was chiefly designed to examine the antiproliferative and antioxidant activities of some novel quinazolinone(thione) derivatives **6**–**14**. The present work focused on two main points; firstly, comparing between quinazolinone and quinazolinthione derivatives. Whereas, antiproliferative (against two cell lines namely, HepG2 and MCF-7) and antioxidant (by two methods; ABTS and DPPH) activities of the investigated compounds, the best quinazolinthione derivatives were **6** and **14,** which exhibited excellent potencies comparable to quinazolinone derivatives **5** and **9**, respectively. Secondly, we compared the activity of four series of Schiff bases which included the quinazolinone moiety (**11a**–**d**). In addition, the antiproliferative and antioxidant activities of the compounds with various aryl aldehyde hydrazone derivatives (**11a**–**d**) analogs were studied. The compounds exhibited potency that increased with increasing electron donating group in *p*-position (OH > OMe > Cl) due to extended conjugated systems. Noteworthy, most of antiproliferative and antioxidant activities results for the tested compounds are consistent with the DFT calculations.

## 1. Introduction

Cancer is the second leading cause of death globally, and the contribution of cancer disease to the overall mortality rate is increasing. Economically, the total annual cost of cancer in 2010 was estimated at approximately US$ 1.16 trillion [1]. So that, more rational design, synthesis, and evaluation of new compounds as anticancer, with higher efficiency is considered as urgent mission in the medicinal chemistry field.

**Figure 1:**
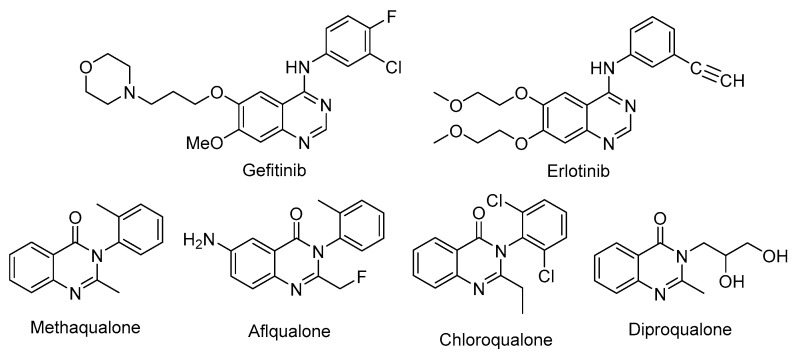
Some structures of synthetic drugs scaffold quinazoline and quinazolinone derivatives.

Quinazoline and quinazolinone derivatives are considered as tremendous targets for the medicinal chemists, due to the fact that they are the scaffold of different potent anticancer drugs, such as Gefitinib (trade name Iressa^®^), Erlotinib (trade name Tarceva^®^) [2,3,4], Methaqualone [5], Afloqualone (as anticonvulsant activity) [6,7], Chloroqualone (as antitussive), and Diproqualone (as sedative-hypnotic agents) [8] (Figure 1).

In the last decades and until now, various compounds including quinazolinone moiety conspicuously exhibited broad spectrum in numerous pharmacological activities such as anticancer [9,10,11,12,13,14], anticonvulsants [15], antiproliferative [16], anti-inflammatory [17], antihypertensive [18], antifungal [19], antibacterial, antioxidant [20], antimicrobial [21], anti-allergic [22], antimalarial [23], antileishmanial [24], and treatment of Alzheimer’s disease (AD) [25].

**Figure 2:**
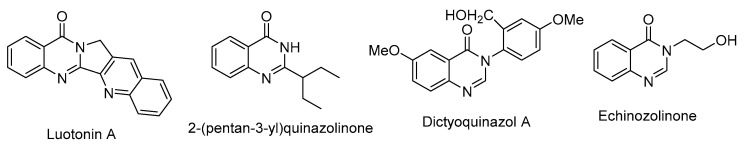
Some structures of naturally occurring quinazolin-4(3*H*)-one derivatives.

Generally, the natural products are considered as one of the most interesting sources of biologically active compounds. Among them, naturally occurring quinazolin-4(3*H*)-one derivatives, which can be isolated from various plants and microorganisms such as Luotonin A (sources; *Peganum nigellastrum*) [26], 2-(heptan-3-yl)quinazolinone (sources; *Bacillus cereus*) [27], Dictyoquinazol A (sources; *Dictyophora indusiata*) [28], and Echinozolinone (sources; *Echinops echinatus*) [29] (Figure 2).

Quinazolinones have been synthesized by different methodologies [28,30,31,32,33,34,35,36,37], in the present study, the conventional methodology to construct novel qunizolinone compounds has been adopted, followed by the study of the antiproliferative activity, antioxidant activity, and DFT calculations for the synthesized compounds.

## 2. Results and Discussion

### 2.1. Chemistry

**Scheme 1:**
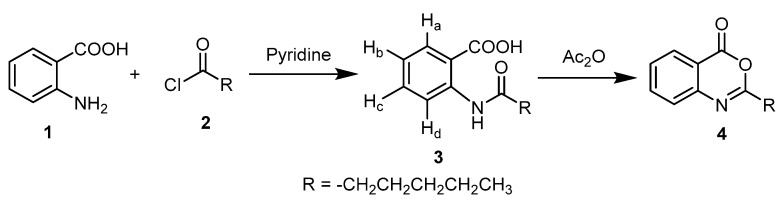
The strategy for synthesis of compound **4**.

In this interesting work, curing of anthranilic acid **1** with hexanoyl chloride **2** in dry pyridine afforded the corresponding *N*-hexanoyl derivative **3** [38], which was cyclized by heating in distilled acetic anhydride to give 2-pentyl-4*H*-benzo[*d*][1,3]oxazin-4-one **4** [39,40] (Scheme 1).

**Scheme 2:**
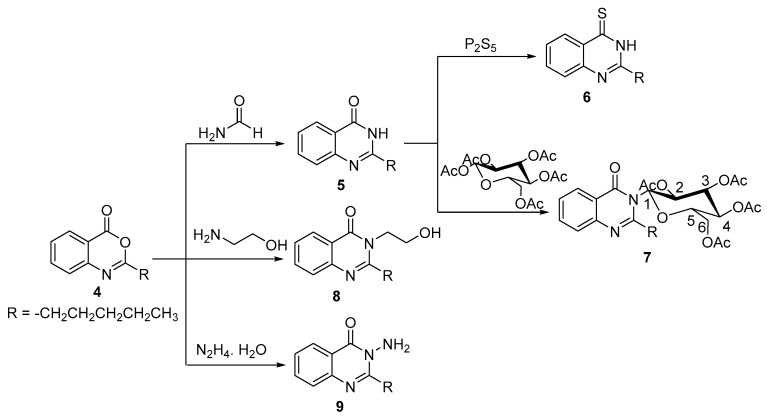
Synthetic route to compounds **5**–**9**.

Benzoxazinone derivative **4** was utilized in situ as a precursor to construct new quinazolinone derivatives. For instance, reaction of benzoxazinone derivative **4** with formamide afforded 2-pentylquinazolin-4(3*H*)-one **5** [41] (Scheme 2). The ^1^H NMR spectrum of **5** exhibited a singlet peak at 12.13 ppm exchangeable with D_2_O corresponding to NH proton, two doublet and two triplet peaks in the aromatic region at 8.05–7.42 ppm corresponding to four aromatic protons, and four characteristic peaks upfield at 2.56–0.84 ppm for *n*-pentyl protons.

Afterwards, sulfuration of 2-pentylquinazolin-4(3*H*)-one **5** by utilizing of phosphorus pentasulfide in dry toluene afforded 2-pentylquinazoline-4(3*H*)-thione **6** (Scheme 2). The formation of compound **6** was unambiguously elaborated by the presence of intense band at 1236 cm^−1^ corresponding to υc=s and the absence of the stretching band of υc=o in the IR spectrum. On the other hand, the incorporation of β-d-glucose pentaacetate with quinazolinone derivative **5** at the nitrogen atom of the later awarded *N*-(β-d-glucopyranosyl-2,3,4,6-tetraacetate)-2-pentyl quinazolin-4(3*H*)-one **7** (Scheme 2), via attacking of the lone pair of nitrogen atom of quinazolinone derivative **5** at the anomeric carbon (C_1_) of β-d-glucose pentaacetate, followed by ring opening and then ring closure with expulsion of acetate as a leaving group.

The chemical structure of compound **7** was explained by the IR spectrum, whereas it showed a band at 1746 cm^−1^ compatible with υ_C=O_ of the acetate groups and lacked the absorption band for the NH group. Moreover, this structure was also interpreted by the ^1^H-NMR spectrum which revealed seven signals at 5.92–3.51 ppm and four singlet signals at 1.97–1.91 ppm all of them corresponding to the protons of β-d-glucopyranosyl-2,3,4,6-tetraacetate moiety. 

Curing of the benzoxazinone derivative **4** with ethanolamine under reflux for 3 h afforded 3-(2-hydroxyethyl)-2-pentylquinazolin-4(3*H*)-one **8** as the sole product. The IR spectrum of compound **8** showed a broad band at 3395 cm^−1^ corresponding to OH functionality. Furthermore, the ^1^H-NMR spectrum appreciably emerged a triplet peak at 4.95 ppm exchangeable with D_2_O corresponding to OH proton, triplet, and quartet peaks at 4.11 and 3.65 ppm, respectively, compatible with ethyl protons of 2-hydroxyethyl moiety. As well, the ^13^C-NMR spectrum exhibited two peaks at 58.8 and 46.1 ppm corresponding to the two carbons of 2-hydroxyethyl moiety. 

3-Amino-2-pentylquinazolin-4(3*H*)-one **9** was commenced by refluxing of compound **4** with hydrazine monohydrate in absolute ethanol for 4 h (Scheme 2). The formation of compound **9** was confirmed by spectroscopic and elemental data. In particular, the ^1^H-NMR spectrum of compound **9** manifested a singlet signal commutable in D_2_O at 5.70 ppm corresponding to NH_2_ protons.

**Scheme 3:**
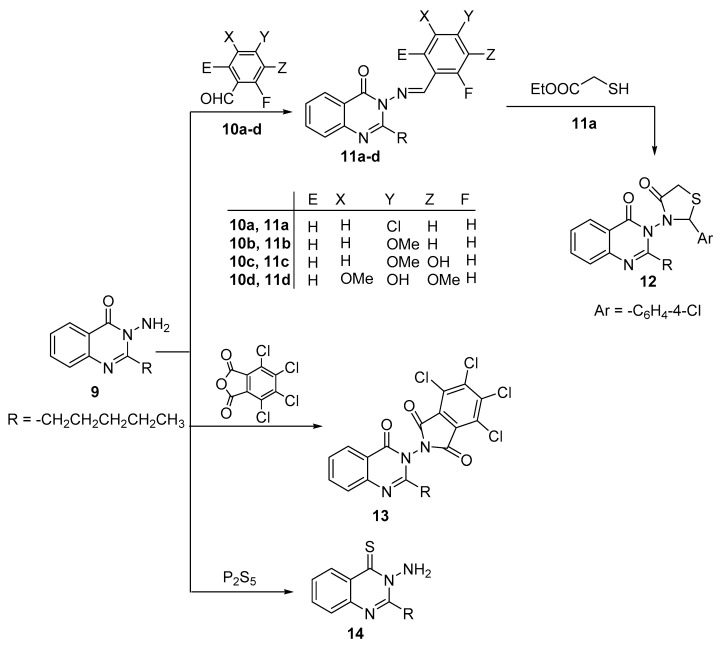
Synthetic route to compounds **11**–**14**.

Reaction of 3-amino-2-pentylquinazolin-4(3*H*)-one **9** with various aldehydes **10a**–**d** gave Schiff bases **11a**–**d** as the sole product in each case (Scheme 3). The ^1^H-NMR spectra of compounds **11a**–**d** exhibited the appearance of a singlet signal in the region between 8.81–8.69 ppm compatible with methine proton of N=CH group. 

The thiazolidin-4-one moiety **12** was constructed by the reaction of Schiff base **11a** with methyl thioglycolate in absolute ethanol including a small amount of piperidine as a catalyst for 3 h (Scheme 3). The prospective structure **12** is in keeping with its spectral and elemental analyses. 

Additionally, the nucleophilicity of the amino group of compound **9** was also estimated by fusion of it with 4,5,6,7-tetrachloroisobenzofuran-1,3-dione in oil bath for an hour and that afforded phthalimido derivative **13** in an excellent yield (Scheme 3). The foreseeable structure of compound **13** was elucidated by their spectral data and elemental analysis. Obviously, its IR spectrum showed stretching absorption bands at 1788, and 1746 cm^−1^ corresponding to the carbonyl groups of phthalimido moiety and at 1707 cm^−1^ corresponding to carbonyl group of the quinazolinone moiety. The ^1^H-NMR spectrum exhibited four peaks for four aromatic protons and another four peaks for n-pentyl protons. Furthermore, its ^13^C-NMR spectrum emerged variant peaks, all of them fit with the proposed structure. 

Eventually, the thione derivative **14** was obtained via sulfuration of compound **9** by utilizing phosphorus pentasulfide as the above pervious method (Scheme 3). The structure of **14** was unequivocally explained via the existence of a peak in the ^13^C NMR spectrum at 182.1 ppm compatible with the carbon of the thione functional group. 

### 2.2. Biological Evaluation

#### 2.2.1. Antiproliferative Screening

Twelve compounds possessing quinazolinone(thione) moieties **5**–**14** along with compound **3** were screened against two cell lines, namely hepatocellular carcinoma (HepG2) and mammary gland (MCF-7) in vitro by utilizing MTT assay [42,43]. The latter assay is a colorimetric test based on the change of the yellow MTT (3-(4,5-dimethylthiazolyl-2)-2,5-diphenyltetrazolium bromide) to a purple formazan derivative by mitochondrial succinate dehydrogenase in viable cells and the Doxorubicin (DOX) was used as a standard reference.

**Figure 3:**
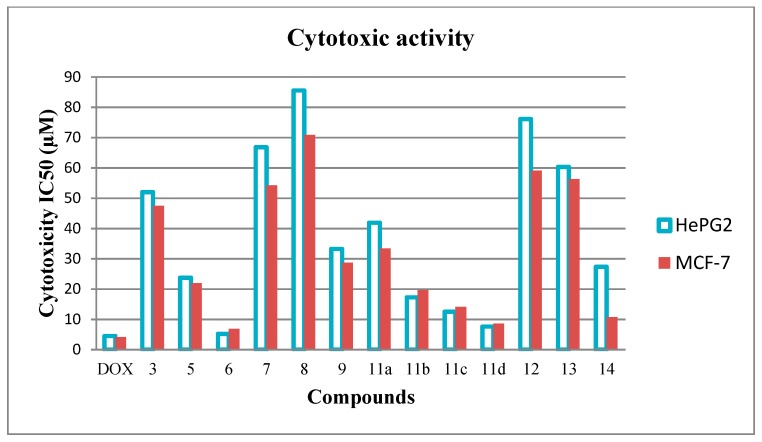
Cytotoxic efficacy of thirteen compounds against HePG2 and MCF-7 cell lines.

The results listed in Table 1 and illustrated in Figure 3, demonstrate that compounds **6** and **11d** have a very strong efficacy against HePG2 cell line with IC_50_ values at 5.20 ± 0.5 and 7.63 ± 0.6 μM, respectively. Meanwhile, compounds **6, 11d,** and **14** have a very strong efficacy against the MCF-7 cell line with IC_50_ values at 6.88 ± 0.4, 8.60 ± 0.7, and 10.78 ± 0.9 μM, respectively. Compounds **11b, 11c** have a strong efficacy against both cell lines with IC_50_ values in the range (12.54 ± 1.1–19.68 ± 1.6 μM). For the HePG2 cell line, compounds **5, 9, 11a,** and **14** have a moderate efficacy with IC_50_ values in the range (23.75 ± 1.9–41.92 ± 2.8 μM). Where, for the MCF-7 cell line, compounds **3, 5, 9,** and **11a** have a moderate efficacy with IC_50_ in the range (21.98 ± 1.8–47.53 ± 2.9 μM). Ultimately, the remaining compounds in both cases have weak efficacies with IC_50_ values > 50 μM.

**Figure 4:**
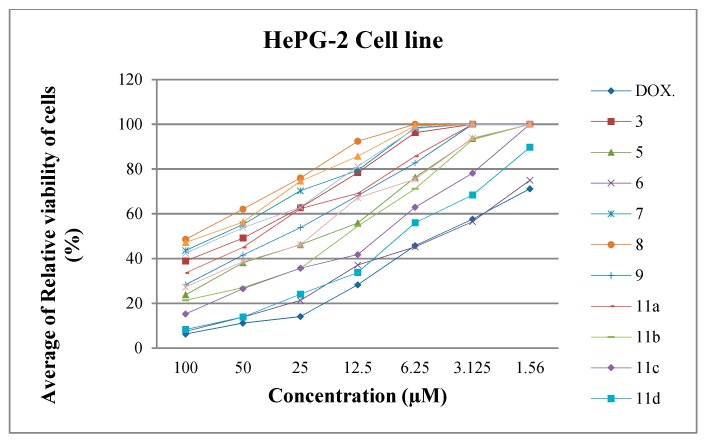
Average of relative viability of HePG-2 cell line (%) with different concentrations.

**Figure 5:**
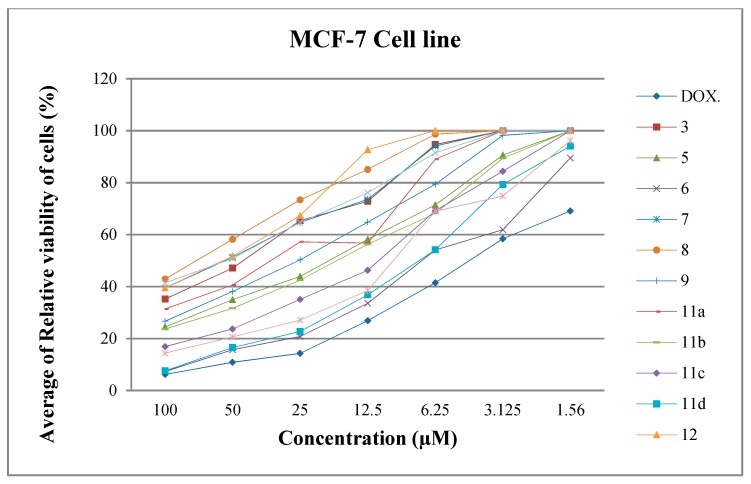
Average of relative viability of MCF-7 cell line (%) with different concentrations.

Through our screening of the antiproliferative efficacy of the synthesized compounds, it was determined that the average of relative viability of cells (%) with different concentrations such as 100, 50, 25, 12.5, 6.25, 3.125, and 1.56 μM against two cell lines (HePG2 and MCF-7) as shown in Figure 4 and Figure 5.

##### Structure Activity Relationship (SAR)

By comparing the antiproliferative efficacy of the thirteen synthesized compounds in this study to their chemical structures, it was concluded that the following structure activity relationship’s (SAR’s) is hypothesized:

1. Conversion of quinazolin-4(3*H*)-one derivative **5** to quinazolin-4(3*H*)-thione derivative **6** enhanced the antiproliferative activity against both cell lines from moderate activity to very strong activity.

2. Similarly, conversion of 3-amino-2-pentylquinazolin-4(3*H*)-one **9** to 3-amino- 2-pentylquinazoline-4(3*H*)-thione **14** enhanced the antiproliferative activity against both cell lines.

3. Reaction of **9** with various aryl aldehydes afforded hydrazone derivatives **(11a**–**d)** analoges with variable potencies according to the following sequence: 3,5-(OMe)_2_-4-OH-C_6_H_2_ **11d** > 3-OH-4-(OMe)-C_6_H_3_ **11c >** 4-(OMe)-C_6_H_4_ **11b >** 4-Cl-C_6_H_4_ **11a**, whereas, the OH group in *p*-position is more electron donating group than the OMe group and Cl atom (i.e., the delocalization of n-π electrons decreased in the above sequence).

4. Construction of the thiazolidinone ring in compound **12** decreased the antiproliferative activity comparable with the hydrazone derivative **11a**, due to decreasing of the delocalization of n-π electrons after replacement of the C=N group (electron attracting group) by the thiazolidinone ring. 

#### 2.2.2. Antioxidant Activity Screening

**Figure 6:**
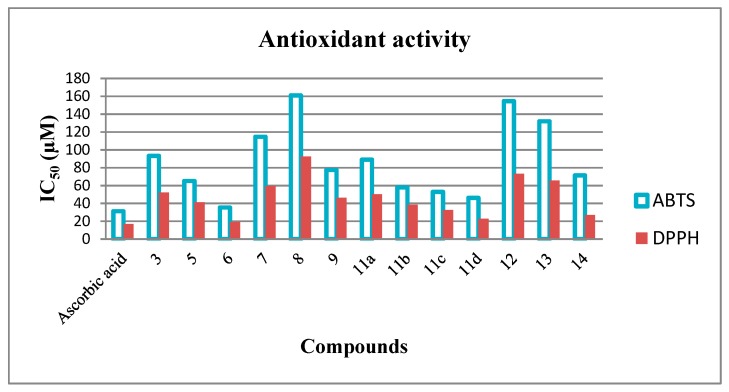
Antioxidant activities of all synthesized compounds by using ABTS and DPPH methods.

One of the aims of this work is the screening of all synthesized compounds for antioxidant activity using two different methods, namely ABTS [2,2′-azino-bis(3-ethyl benzothiazoline-6-sulfonic acid)] and DPPH assays. DPPH (2,2-diphenyl-1-picryl-hydrazyl-hydrate) free radical assay based on electron-transfer that produces a violet solution in ethanol. This free radical is stable at ambient temperature and reduced in the presence of an antioxidant molecule, leading to colorless ethanol solution. After investigation of these results as listed in Table 2 and Figure 6, it was realized that, compounds **6** and **11d** have promising activity through using ABTS assay. Meanwhile, in the case of using DPPH assay, compounds **6**, **11d** and **14** have also very high activity. Ascorbic acid was used as a reference through the antioxidant activity screening.

The results depicted in Table 2 and Figure 6 demonstrated that, DPPH assay findings are very approximately related to those of ABTS assay with only one exception, compound **14** has an excellent antioxidant activity against DPPH (IC_50_ = 26.87 ± 0.23 μM) than that of the ABTS method (IC_50_ = 71.42 ± 0.52 μM). Noteworthy, all the screened compounds in the case of the DPPH method exhibited IC_50_ smaller than the corresponding ones of the same compounds in the case of the ABTS method, and it proposed that these compounds are more promising scavengers of the DPPH radical than those of the ABTS radical.

By comparing the antioxidant efficacy of the thirteen synthesized compounds in this study to their chemical structures, it was concluded that the following structure antioxidant activity relationship’s (SAR’s) is hypothesized:

1. The presence of C=S enhanced antioxidant activity than the presence of C=O, as shown in compounds **6** and **14** comparable with compounds **5** and **9**, respectively.

2. The hydrazone derivatives **(11a**–**d)** analogs have variable potencies according to the following sequence: 3,5-(OMe)_2_-4-OH-C_6_H_2_ **11d** > 3-OH-4-(OMe)-C_6_H_3_ **11c** > 4-(OMe)-C_6_H_4_ **11b** > 4-Cl-C_6_H_4_ **11a**, whereas, OH group in *p*-position is a more electron donating group (has more conjugated system) than OMe group and Cl atom.

3. In compound **12**, replacement of C=N group by the thiazolidinone ring decreased the antioxidant activity comparable with **11a**, because of the lack of the conjugated system.

Previous reports of structurally similar compounds (in quinazoline ring) but with different substituents have demonstrated different results in antiproliferative and antioxidant activities from our results [9,33,44].

### 2.3. Density Functional Theory

According to the frontier molecular orbital (FMO) theory, the highest occupied molecular orbital (HOMO) acts as an electron-donor and the lowest unoccupied molecular orbital (LUMO) acts as an electron-acceptor [45]. Meanwhile, both play remarkable roles in the electronic studies by using quantum chemical calculations and they are also of significant importance in modern biochemistry and molecular biology [46]. A molecule is considered as a softer and has an excellent chemical reactivity when it has a smaller energy gap. Meanwhile, a molecule is considered to have a higher chemical hardness and assumed to have good stability when it has a larger energy gap [47,48,49,50,51].

The quantum chemical calculations were implemented by the density functional theory (DFT) method by using the Gaussian(R) 09 program at the B3LYP level in conjunction with 6-31G(d,p) basis set and computed parameters are summarized in Table 3.

By computing and using the energy gap (∆*E* = *E*_LUMO_ − *E*_HOMO_) and dipole moment values beside another quantum chemical parameters such as ionization energy (*I* = −*E*_HOMO_), electron affinity (*A* = −*E*_LUMO_) [52], chemical hardness (*η* = (I − A)/2), chemical softness (S = 1/*η*) [53], and binding energy, we can rationally explicate the relation between the chemical structure and the antiproliferative activity (SAR’s). Whereas, the energy gap of compound **6** (∆*E* = 3.98 eV) is smaller than that corresponding of compound **5** (∆*E* = 4.85 eV) and also compound **6** has a higher chemical softness value (*S* = 0.50 eV^−1^) than that corresponding of compound **5** (*S* = 0.41 eV^−1^). These results are matching with the results of the antiproliferative screening whereas, compound **6** has a higher potency comparable with compound **5** for both cell lines (HepG2 and MCF-7) as shown in Table 1 and Figure 3. Similarly, compound **14** has a smaller energy gap and a higher chemical softness than that corresponding to compound **9** as listed in Table 2. In addition, in vitro compound **14** showed a remarkable higher efficacy comparable with compound **9** as shown in Table 1 and Figure 3. Notably, the dipole moment values of compounds **6** (µ = 3.4641 D) and **14** (µ = 1.852 D) are higher than that of compounds **5** (µ = 3.2867 D) and **9** (µ = 1.764 D), respectively.

On the other hand, compounds **11a**–**d** possess antiproliferative activity in the following order **11d** > **11c** > **11b** > **11a**, meanwhile, the energy gaps of these compounds increase in the following order **11a** (∆*E* = 2.99 eV) < **11d** (∆*E* = 3.05 eV) < **11c** (∆*E* = 3.10 eV) < **11b** (∆*E* = 3.13 eV). The lower of the antiproliferative activity of compound **11a** may be explained by values of the dipole moment whereas; the dipole moment of compound **11a** is smaller than that of compounds **11b**–**d** as shown in Table 3. 

**Figure 7:**
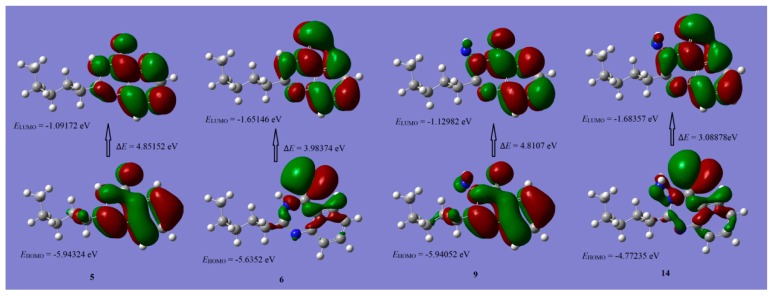
Schematic representation of HOMO and LUMO coefficient distribution of compounds **5, 6, 9**, and **14**.

**Figure 8:**
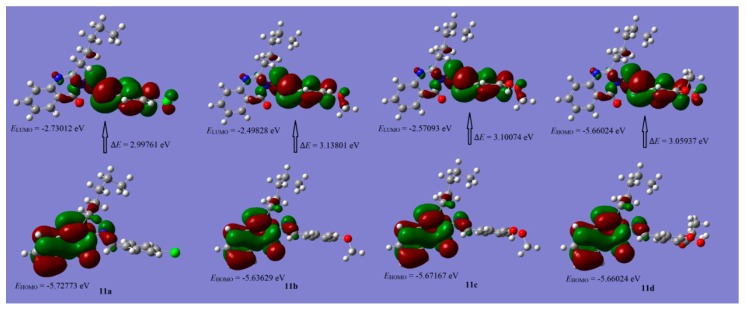
Schematic representation of highest occupied molecular orbital (HOMO) and loest unoccupied molecular orbital (LUMO) coefficient distribution of compounds **11a**–**d**.

The distributions of the HOMO and LUMO orbitals of the selected compounds are computed at the same level of the DFT theory and are provided in Figure 7 and Figure 8. The results manifested that possible reactive sites exist as shown below:

1. The HOMO of compounds **5** and **9** are nearly similar and the distribution of orbitals are mainly situated on the quinazolinone moiety, also, the LUMO of these compounds are situated on the same moiety.

2. The HOMO of compounds **6** and **14** are nearly similar and the distribution of orbitals are mainly situated on C=S, while, the LUMO of these compounds are mainly situated on the quinazolinthione moiety.

3. The HOMO of compounds **11a**–**d** are nearly similar and the distribution of orbitals are mainly situated on the quniazolinone moiety, meanwhile, the LUMO of these compounds are mainly situated on the aryl aldehyde hydrazone system.

## 3. Materials and Methods

### 3.1. Chemistry

The melting point is uncorrected and was measured on a Stuart SMP 30 advanced digital electric melting point apparatus (Cole-Parmer, Staffordshire, UK). All reactions were monitored by TLC (Kieselgel 60 F_254_, Merck, Munchen, Germany) and spots were visualized using UV (254 nm), In the region (400−4000 cm^−1^), the IR spectrum was measured in the KBr phase by using the Nicolet iS10 FT-IR spectrometer (Shimadzu Corporation, Kyoto, Japan). The ^1^H-NMR (at 400 MHz) and ^13^C-NMR (at 100 MHz) spectra were performed at chemical warfare labs, Egypt, with a Varian Gemini spectrometer (Metrohim, California, United States) in DMSO-*d*_6_ as a solvent by using tetramethylsilane (TMS) as a reference. Perkin-Elmer 2400 CHN elemental analyzer (Waltham, MA, USA) was used to record CHN elemental analysis at the Faculty of Science, Cairo University, Egypt. The mass spectrum was measured on Shimadzu GC-MS QP1000EX apparatus (Shimadzu Corporation, Kyoto, Japan) at the central analytical lab, Ain Shams University, Cairo, Egypt. 

#### 3.1.1. 2-Hexanamidobenzoic Acid **3**

Hexanoyl chloride **2** (1.39 mL, 0.01 mol) was added dropwise to anthranilic acid **1** (1.37 g, 0.01 mol) dissolved in dry pyridine (20 mL) at ambient temperature with stirring. The stirring was continued for an hour, and then the resulting emulsion was acidified with cold 10% HCl solution. The white solid which separated was collected by filtration and then recrystallized from benzene to give **3** [38] as white crystals; m.p.: 92–95 °C (Lit. m.p.: 93−95 °C) [38], yield: 92%. IR (KBr, cm^−1^): 3426−2463 (br) (OH), 3206 (NH), 2959, 2934, 2861 (CH_aliph._), 1691, 1637 (C=O). ^1^H-NMR (400 MHz, DMSO-*d*_6_) δ (ppm): 13.51 (br.s, 1H, OH, exchangeable with D_2_O), 11.09 (s, 1H, NH, exchangeable with D_2_O), 8.48 (d, 1H, Ar-H, H_a_, *J* = 8.8 Hz), 7.95 (d, 1H, Ar-H, H_d_, *J* = 8.0 Hz), 7.55 (t, 1H, Ar-H, H_c_, *J* = 7.8 Hz, *J* = 8.0 Hz), 7.11 (t, 1H, Ar-H, H_b_, *J* = 7.4 Hz, *J* = 7.8 Hz), 2.35 (t, 2H, COCH_2_, *J* = 7.2 Hz, *J* = 7.6 Hz), 1.60 (quintet, 2H, COCH_2_CH_2_, *J* = 7.2 Hz, *J* = 7.6 Hz), 1.31−1.26 (m, 4H, CH_3_CH_2_CH_2_), 0.85 (t, 3H, CH_3_, *J* = 6.8 Hz, *J* =7.2 Hz), MS *m*/*z* (%): 235 (M^.+^; 29.4). Anal. Calcd. for C_13_H_17_NO_3_ (235.28): C, 66.36; H, 7.28; N, 5.95. Found: C, 66.36; H, 7.28; N, 5.95. 

#### 3.1.2. 2-Pentyl-4*H*-benzo[*d*][1,3]oxazin-4-one **4**

A suspension of 2-hexanamidobenzoic acid **3** (2.35 g, 0.01 mol) in freshly distilled acetic anhydride (10 mL) was heated in water bath for an hour followed by a concentration of the mixture in vacuo and used in situ [39,40].

#### 3.1.3. 2-Pentylquinazolin-4(3*H*)-one **5**

A solution of benzoxazinone **4** (2.17 g, 0.01 mol) in formamide (15 mL) was refluxed for 7 h. After cooling, the reaction mixture was poured onto ice cold water, the obtained solid was filtered off, dried, and recrystallized from petroleum ether 60–80 °C to give **5** [41] as white crystals; m.p.: 142–144 °C (Lit. m.p.: 153–154 °C) [41], yield: 92%. IR (KBr, cm^−1^): 3171 (NH), 2958, 2928, 2860 (CH_aliph._), 1680 (C=O), 1614 (C=N or C=C). ^1^H-NMR (400 MHz, DMSO-*d*_6_) δ (ppm): 12.13 (s, 1H, NH, exchangeable with D_2_O), 8.05 (d, 1H, Ar-H, H_a_, *J* = 8.0 Hz), 7.74 (t, 1H, Ar-H, H_c_, *J* = 7.6 Hz, *J* = 7.8 Hz), 7.56 (d, 1H, Ar-H, H_d_, *J* = 8 Hz), 7.42 (t, 1H, Ar-H, H_b_, *J* = 7.6 Hz), 2.56 (t, 2H, N=CCH_2_, *J* = 7.6 Hz, *J* = 8.0 Hz), 1.70 (quintet, 2H, N=CCH_2_CH_2_, *J* = 7.6 Hz, *J* = 7.2 Hz), 1.30–1.26 (m, 4H, CH_3_CH_2_CH_2_), 0.84 (t, 3H, CH_3_, *J* = 6.8 Hz, *J* = 7.2 Hz). ^13^C-NMR (100 MHz, DMSO-*d*_6_) δ (ppm): 162.2, 157.9, 149.4, 134.7, 127.2, 126.3, 126.1, 121.2, 34.9, 31.1, 26.9, 22.2, 14.2. MS *m*/*z* (%): 216 (M^.+^; 26.3). Anal. Calcd. for C_13_H_16_N_2_O (216.28): C, 72.19; H, 7.46; N, 12.95. Found: C, 72.26; H, 7.49; N, 12.86. 

#### 3.1.4. 2-Pentylquinazoline-4(3*H*)-thione **6**

To a solution of quinazolinone **5** (2.16 g, 0.01 mol) in dry toluene (30 mL), P_2_S_5_ (2.22 g, 0.01 mol) was added. The reaction mixture was refluxed for 1 h, and then filtered off. The obtained filtrate was evaporated under reduced pressure, the formed solid was collected by filtration, dried, and recrystallized from ethanol to give **6** as light brown crystals; m.p.: 101–103 °C, yield: 76%. IR (KBr, cm^−1^): 3184, 3141 (NH), 2967, 2935, 2852 (CH_aliph._), 1618 (C=N), 1604 (C=C), 1236 (C=S). ^1^H-NMR (400 MHz, DMSO-*d*_6_) δ (ppm): 13.69 (s, 1H, NH, exchangeable with D_2_O), 8.52 (d, 1H, Ar-H, H_d_, *J* = 8.4 Hz), 7.83 (t, 1H, Ar-H, H_c_, *J* = 7.6 Hz), 7.63 (d, 1H, Ar-H, H_a_, *J* = 8.4 Hz), 7.52 (t, 1H, Ar-H, H_b_, *J* = 7.4 Hz, *J* = 7.8 Hz), 2.70 (t, 2H, N=CCH_2_, *J* = 7.6 Hz, *J* = 8.0 Hz), 1.72 (quintet, 2H, N=CCH_2_CH_2_, *J* = 7.6 Hz, *J* = 7.2 Hz), 1.31−1.26 (m, 4H, CH_3_CH_2_CH_2_), 0.85 (t, 3H, CH_3_, *J* = 6.8 Hz). MS *m*/*z* (%): 232 (M^.+^; 16.7). Anal. Calcd. for C_13_H_16_N_2_S (232.35): C, 67.20; H, 6.94; N, 12.06; S, 13.80. Found: C, 67.31; H, 7.03; N, 12.01; S, 13.85. 

#### 3.1.5. *N*-(β-d-Glucopyranosyl-2,3,4,6-tetraacetate)-2-pentylquinazolin-4(3H)-one **7**

Quinazolinone **5** (2.16 g, 0.01 mol) was refluxed with β-d-glucose pentaacetate (3.90 g, 0.01 mol) in absolute ethanol (50 mL) for 3 h. the solid obtained after slow evaporation of the resulting solution was collected and recrystallized from ethanol to give **7** as white crystals; m.p.: 135–137 °C, yield: 62%. IR (KBr, cm^−1^): 2955, 2924, 2854 (CH_aliph._), 1746 (C=O_ester_), 1678 (C=O_quinazolinone_), 1613 (C=N). ^1^H-NMR (400 MHz, DMSO-*d*_6_) δ (ppm): 8.05 (d, 1H, Ar-H, H_a_, *J* = 8.0 Hz), 7.74 (t, 1H, Ar-H, H_c_, *J* = 8.4 Hz, *J* = 6.8 Hz), 7.56 (d, 1H, Ar-H, H_d_, *J* = 8.4 Hz), 7.43 (t, 1H, Ar-H, H_b_, *J* = 8.0 Hz, *J* = 7.2 Hz), 5.92 (d, 1H, C_1_-H, *J* = 8.4 Hz), 5.39 (t, 1H, C_2_-H, *J* = 9.6 Hz), 4.93 (t, 1H, C_3_-H, *J* = 9.6 Hz), 4.90 (t, 1H, C_4_-H, *J* = 8.4 Hz, *J* = 10.0 Hz), 4.14, 412 (d,d, 1H, C_6_-H, *J* = 10.4 Hz, *J* = 5.6 Hz), 3.97 (d, 1H, C_6_-H, *J* = 10.4 Hz), 3.51 (m, 1H, C_5_-H), 2.56 (t, 2H, N=CCH_2_, *J* = 8.0 Hz, *J* = 7.6 Hz), 1.978 (s, 3H, CH_3_), 1.973 (s, 3H, CH_3_), 1.961 (s, 3H, CH_3_), 1.917 (s, 3H, CH_3_), 1.69 (quintet, 2H, N=CCH_2_CH_2_, *J* = 7.6 Hz, *J* = 6.8 Hz), 1.31−1.26 (m, 4H, CH_3_CH_2_CH_2_), 0.84 (t, 3H, CH_3_, *J* = 6.8 Hz, *J* = 7.2 Hz). MS *m*/*z* (%): 546 (M^+^; 32.4). Anal. Calcd. for C_27_H_34_N_2_O_10_ (546.57): C, 59.33; H, 6.27; N, 5.13. Found: C, 59.18; H, 6.21; N, 5.08.

#### 3.1.6. 3-(2-Hydroxyethyl)-2-pentylquinazolin-4(3*H*)-one **8**

A solution of benzoxazinone **4** (2.17 g, 0.01 mol) in ethanolamine (15 mL) was heated under reflux for 3 h. The reaction mixture was poured onto ice cold water, the obtained solid was filtered off, dried, and then recrystallized from ethanol to give **8** as white crystals; m.p.: 84–85 °C, yield: 47%. IR (KBr, cm^−1^): 3395 (OH), 2953, 2931, 2872 (CH_aliph._), 1648 (C=O), 1611 (C=N). ^1^H-NMR (400 MHz, DMSO-*d*_6_) δ (ppm): 8.07 (d, 1H, Ar-H, H_a_, *J* = 8.0 Hz), 7.75 (t, 1H, Ar-H, H_c_, *J* = 7.8 Hz, *J* = 7.6 Hz), 7.57 (d, 1H, Ar-H, H_d_, *J* = 8.0 Hz), 7.44 (t, 1H, Ar-H, H_b_, *J* = 7.6 Hz, *J* = 7.2 Hz), 4.95 (t, 1H, OH, exchangeable with D_2_O, *J* = 5.6 Hz), 4.11 (t, 2H, CH_2_CH_2_OH, *J* = 5.6 Hz, *J* = 6.0 Hz), 3.65 (q, 2H, CH_2_OH, *J* = 6.0 Hz, *J* = 5.6 Hz), 2.93 (t, 2H, N=CCH_2_, *J* = 7.6 Hz, *J* = 8.0 Hz), 1.75 (quintet, 2H, N=CCH_2_CH_2_, *J* = 7.6 Hz, *J* = 7.6 Hz), 1.40−1.32 (m, 4H, CH_3_CH_2_CH_2_), 0.88 (t, 3H, CH_3_, *J* = 7.2 Hz). ^13^C-NMR (100 MHz, DMSO-*d*_6_) δ (ppm): 161.7, 158.4, 147.4, 134.6, 127.1, 126.5, 126.4, 120.4, 58.8, 46.1, 34.6, 31.3, 26.3, 22.4, 14.3. MS *m*/*z* (%): 260 (M^+^; 41.2). Anal. Calcd. for C_15_H_20_N_2_O_2_ (260.34): C, 69.20; H, 7.74; N, 10.76. Found: C, 69.17; H, 7.68; N, 10.81. 

#### 3.1.7. 3-Amino-2-pentylquinazolin-4(3*H*)-one **9**

A mixture of benzoxazinone **4** (2.17 g, 0.01 mol) and hydrazine hydrate (1.5 mL) in absolute ethanol (20 mL) was refluxed for 3 h. The mixture was poured onto ice cold water, the formed solid was filtered off, and recrystallized from ethanol to give **9** as buff crystals; m.p.: 58–60 °C, yield: 43%. IR (KBr, cm^−1^): 3306, 3263 (NH_2_), 2954, 2931, 2910, 2856 (CH_aliph._), 1673 (C=O), 1630 (C=N). ^1^H NMR (400 MHz, DMSO-*d*_6_) δ (ppm): 8.08 (d, 1H, Ar-H, H_a_, *J* = 7.8 Hz), 7.75 (t, 1H, Ar-H, H_c_, *J* = 7.4 Hz, *J* = 7.8 Hz), 7.59 (d, 1H, Ar-H, H_d_, *J* = 7.6 Hz), 7.45 (t, 1H, Ar-H, H_b_, *J* = 7.2 Hz, *J* = 7.6 Hz), 5.70 (s, 2H, NH_2_, exchangeable with D_2_O), 2.90 (t, 2H, N=CCH_2_, *J* = 7.2 Hz, *J* = 8.0 Hz), 1.74 (quintet, 2H, N=CCH_2_CH_2_, *J* = 7.6 Hz, *J* = 7.2 Hz), 1.37−1.30 (m, 4H, CH_3_CH_2_CH_2_), 0.87 (t, 3H, CH_3_, *J* = 6.4 Hz, *J* =7.2 Hz). ^13^C-NMR (400 MHz, DMSO-d_6_) δ (ppm): 160.9, 158.8, 147.0, 134.3, 127.2, 126.39, 126.31, 120.2, 34.0, 31.4, 26.0, 22.3, 14.3. MS *m*/*z* (%): 231 (M^+^; 41.1). Anal. Calcd. for C_13_H_17_N_3_O (231.30): C, 67.51; H, 7.41; N, 18.17. Found: C, 67.39; H, 7.34; N, 18.24.

#### 3.1.8. General Procedure for Synthesis of **11a**–**d**

A mixture of compound **9** (2.31 g, 0.01 mol) and the appropriate aldehydes **10a**–**d** (0.01 mol) in absolute ethanol (30 mL) was refluxed for 4−6 h. The reaction mixture was evaporated under reduced pressure; the obtained residue was collected and recrystallized from the proper solvent to give the corresponding benzylidene derivatives **11a**–**d**, respectively.

##### 3-((4-Chlorobenzylidene)amino)-2-pentylquinazolin-4(3*H*)-one **11a**

Yellow crystals; m.p.: 176–178 °C (ethanol), yield: 72%. IR (KBr, cm^−1^): 2943, 2866 (CH_aliph._), 1667 (C=O), 1624 (C=N). ^1^H-NMR (400 MHz, DMSO-*d*_6_) δ (ppm): 8.69 (s, 1H, N=CH), 7.95 (d, 1H, Ar-H, H_a_, *J* = 7.8 Hz), 7.88 (d, 2H, Ar-H, H_E_ + H_F_, *J* = 8.8 Hz), 7.66 (t, 1H, Ar-H, H_c_, *J* = 8.4 Hz), 7.57 (d, 3H, Ar-H, H_d_ + H_X_ + H_Z_), 7.46 (t, 1H, Ar-H, H_b_, *J* = 8.4 Hz), 2.34 (t, 2H, N=CCH_2_, *J* = 7.6 Hz), 1.60 (quintet, 2H, N=CCH_2_CH_2_, *J* = 7.2 Hz), 1.31−1.26 (m, 4H, CH_3_CH_2_CH_2_), 0.85 (t, 3H, CH_3_, *J* = 6.8 Hz). MS *m*/*z* (%): 353 (M^+^; 4.0). Anal. Calcd. for C_20_H_20_ClN_3_O (353.85): C, 67.89; H, 5.70; Cl, 10.02; N, 11.88. Found: C, 67.78; H, 5.62; Cl, 9.89; N, 11.79.

##### 3-((4-Methoxybenzylidene)amino)-2-pentylquinazolin-4(3*H*)-one **11b**

White crystals; m.p.: 83–84 °C (petroleum ether 60–80 °C), yield: 64%. IR (KBr, cm^−1^): 2946, 2912, 2882, 2843 (CH_aliph._), 1669 (C=O), 1606 (C=N or C=C). ^1^H-NMR (400 MHz, DMSO-*d*_6_) δ (ppm): 8.81 (s, 1H, N=CH), 8.12 (d, 1H, Ar-H, H_a_, *J* = 7.8 Hz), 7.89 (d, 2H, Ar-H, H_E_ + H_F_, *J* = 8.8 Hz), 7.80 (t, 1H, Ar-H, H_c_, *J* = 8.2, Hz, *J* = 7.4 Hz), 7.65 (d, 1H, Ar-H, H_d_, *J* = 8.0 Hz), 7.50 (t, 1H, Ar-H, H_b_, *J* = 7.6, Hz, *J* = 7.4 Hz), 7.12 (d, 2H, Ar-H, H_X_ + H_Z_, *J* = 8.8 Hz), 3.85 (s, 3H, OCH_3_), 2.80 (t, 2H, N=CCH_2_, *J* = 7.6 Hz, *J* = 8.0 Hz), 1.71 (quintet, 2H, N=CCH_2_CH_2_, *J* = 7.6 Hz, *J* = 7.2 Hz), 1.33−1.26 (m, 4H, CH_3_CH_2_CH_2_), 0.81 (t, 3H, CH_3_, *J* = 7.2 Hz). MS *m*/*z* 7(%): 349 (M^+^; 11.). Anal. Calcd. for C_21_H_23_N_3_O_2_ (349.43): C, 72.18; H, 6.63; N, 12.03. Found: C, 72.29; H, 6.69; N, 11.88.

##### 3-((3-Hydroxy-4-methoxybenzylidene)amino)-2-pentylquinazolin-4(3*H*)-one **11c**

White crystals; m.p.: 150–152 °C (ethanol), yield: 57%. IR (KBr, cm^−1^): 3277 (OH), 2956, 2927, 2892, 2863, 2845 (CH_aliph._), 1678 (C=O), 1603 (C=N or C=C). ^1^H-NMR (400 MHz, DMSO-*d*_6_) δ (ppm): 9.49 (s, 1H, OH, exchangeable with D_2_O), 8.70 (s, 1H, N=CH), 8.12 (d, 1H, Ar-H, H_a_, *J* = 8.0 Hz), 7.79 (t, 1H, Ar-H, H_c_, *J* = 8.0 Hz, *J* =8.4 Hz), 7.65 (d, 1H, Ar-H, H_d_, *J* = 7.6 Hz), 7.49 (t, 1H, Ar-H, H_b_, *J* = 8.0 Hz, *J* = 7.2 Hz), 7.42 (d, 1H, H_F_, *J_m_* = 2 Hz), 7.30, 7.28 (d,d, 1H, Ar-H, H_E_, *J_o_* = 8.4 Hz, *J_m_* = 2 Hz), 7.07 (d, 1H, Ar-H, H_X_, *J* = 8.4 Hz), 3.85 (s, 3H, OCH_3_), 2.78 (t, 2H, N=CCH_2_, *J* = 7.6 Hz), 1.71 (quintet, 2H, N=CCH_2_CH_2_, *J* = 7.6 Hz, *J* = 7.2 Hz), 1.31−1.28 (m, 4H, CH_3_CH_2_CH_2_), 0.81 (t, 3H, CH_3_, *J* = 6.8 Hz, *J* = 7.2 Hz). MS *m*/*z* (%): 365 (M^+^; 23.4). Anal. Calcd. for C_21_H_23_N_3_O_3_ (365.43): C, 69.02; H, 6.34; N, 11.50. Found: C, 68.88; H, 6.28; N, 11.62.

##### 3-((4-Hydroxy-3,5-dimethoxybenzylidene)amino)-2-pentylquinazolin-4(3*H*)-one **11d**

White crystals; m.p.: 148–150 °C (benzene), yield: 61%. IR (KBr, cm^−1^): 3408 (OH), 2952, 2911, 2844 (CH_aliph._), 1668 (C=O), 1591 (C=N or C=C). ^1^H-NMR (400 MHz, DMSO-d_6_) δ (ppm): 9.36 (br.s, 1H, OH, exchangeable with D_2_O), 8.72 (s, 1H, N=CH), 8.12 (d, 1H, Ar-H, H_a_, *J* = 8.2 Hz), 7.79 (t, 1H, Ar-H, H_c_, *J* = 8.0 Hz, *J* = 7.4 Hz), 7.65 (d, 1H, Ar-H, H_d_, *J* = 7.6 Hz), 7.50 (t, 1H, Ar-H, H_b_, *J* = 8.0 Hz, *J* = 7.2 Hz), 7.23 (s, 2H, H_E_ + H_F_), 3.82 (s, 6H, 2OCH_3_), 2.81 (t, 2H, N=CCH_2_, *J* = 7.6 Hz, *J* = 8.0 Hz), 1.72 (quintet, 2H, N=CCH_2_CH_2_, *J* = 7.6 Hz, *J* = 7.2 Hz), 1.36−1.26 (m, 4H, CH_3_CH_2_CH_2_), 0.81 (t, 3H, CH_3_, *J* = 6.8 Hz, *J* =7.2 Hz). ^13^C-NMR (100 MHz, DMSO-*d*_6_) δ (ppm): 169.8, 158.0, 156.5, 148.6 (2), 146.7, 140.8, 134.6, 127.4, 127.0, 126.7, 122.8, 121.3, 106.8 (2), 56.5 (2), 34.5, 31.3, 26.0, 22.2, 14.2. MS *m*/*z* (%): 395 (M^+^; 62.1), Anal. Calcd. for C_22_H_25_N_3_O_4_ (395.46): C, 66.82; H, 6.37; N, 10.63. Found: C, 66.95; H, 6.41; N, 10.58.

#### 3.1.9. 2-(4-Chlorophenyl)-3-(4-oxo-2-pentylquinazolin-3(4*H*)-yl)thiazolidin-4-one **12**

A mixture of compound **11a** (3.53 g, 0.01 mol) and methyl thioglycolate (0.89 mL, 0.01 mol) in absolute ethanol (30 mL) containing piperidine (0.5 mL) was refluxed for 3 h. The obtained solid after evaporation of the solvent was collected and recrystallized from petroleum ether 60–80 °C to give **12** as pale yellow crystals; m.p.: 78–80 °C, yield: 47%. IR (KBr, cm^−1^): 2951, 2925, 2868 (CH_aliph._), 1736 (C=O_thiazolidinone_), 1671 (C=O_quinazolinone_), 1608 (C=N or C=C). ^1^H-NMR (100 MHz, DMSO-*d*_6_) δ (ppm): 8.13 (d, 1H, Ar-H, H_a_, *J* = 8.2 Hz), 7.96 (d, 2H, Ar-H, H_E_ + H_F_, *J* = 8.0 Hz), 7.80 (t, 1H, Ar-H, H_c_, *J* = 7.6 Hz), 7.65 (d, 1H, Ar-H, H_d_, *J* = 8.0 Hz), 7.64 (d, 2H, Ar-H, H_X_ + H_Z_, *J* = 8.4 Hz), 7.51 (t, 1H, Ar-H, H_b_, *J* = 7.6 Hz, *J* = 7.8 Hz), 5.70 (s, 1H, SCH), 3.75, 3.67 (d,d, 2H, CH_2(thiazolidinone)_, *J* = 23.6 Hz, *J* = 23.2 Hz), 2.81 (t, 2H, N=CCH_2_, *J* = 8.0 Hz, *J* = 7.6 Hz), 1.71 (quintet, 2H, N=CCH_2_CH_2_, *J* = 7.6 Hz, *J* = 8.0 Hz), 1.35−1.17 (m, 4H, CH_3_CH_2_CH_2_), 0.80 (t, 3H, CH_3_, *J* = 7.2 Hz). MS *m*/*z* (%): 427 (M^+^; 11.8). Anal. Calcd. for C_22_H_22_ClN_3_O_2_S (427.95): C, 61.75; H, 5.18; Cl, 8.28; N, 9.82; S, 7.49. Found: C, 61.66; H, 5.12; Cl, 8.31; N, 9.75; S, 7.55.

#### 3.1.10. 4,5,6,7-Tetrachloro-2-(4-oxo-2-pentylquinazolin-3(4*H*)-yl)isoindoline-1,3-dione **13**

Compound **9** (2.31 g, 0.01 mol) was fused with 4,5,6,7-tetrachloroisobenzofuran-1,3-dione (2.85 g, 0.01 mol) in oil bath for an hour. The resulting solid was recrystallized from ethanol to give **13** as orange crystals; m.p.: 178–180 °C, yield: 86%. IR (KBr, cm^−1^): 2943, 2856 (CH_aliph._), 1788, 1746 (C=O_imide_), 1705 (C=O_quinazolinone_), 1606 (C=N or C=C). ^1^H-NMR (400 MHz, DMSO-*d*_6_) δ (ppm): 8.08 (d, 1H, Ar-H, H_a_, *J* = 8 Hz), 7.94 (t, 1H, Ar-H, H_c_, *J* = 8.0 Hz, *J* = 7.2 Hz), 7.76 (d, 1H, Ar-H, H_d_, *J* = 8.0 Hz), 7.60 (t, 1H, Ar-H, H_b_, *J* = 7.6 Hz), 2.73 (t, 2H, N=CCH_2_, *J* = 6.8 Hz, *J* = 8.0 Hz), 1.70−1.60 (m, 2H, N=CCH_2_CH_2_), 1.29−1.27 (m, 4H, CH_3_CH_2_CH_2_), 0.81 (t, 3H, CH_3_, *J* = 6.8 Hz, *J* =7.2 Hz). ^13^C-NMR (100 MHz, DMSO-*d*_6_) δ (ppm): 161.5, 161.3, 157.9, 157.7, 147.4, 146.7, 140.0, 138.8, 136.6 (2), 128.2 (2), 128.0, 127.1, 126.8, 119.8, 32.6, 30.9, 26.0, 22.2, 14.2. MS *m*/*z* (%): 231 (M^+^; 41.1). Anal. Calcd. for C_21_H_15_C_l4_N_3_O_3_ (499.17): C, 50.53; H, 3.03; Cl, 28.41; N, 8.42. Found: C, 50.61; H, 3.09; Cl, 28.37; N, 8.53.

#### 3.1.11. 3-Amino-2-pentylquinazoline-4(3*H*)-thione **14**

A mixture of compound **9** (2.31 g, 0.01 mol) and P_2_S_5_ (2.22 g, 0.01 mol) in dry toluene (15 mL) was heated under reflux for 4 h. The mixture was filtered off, the filtrate was evaporated under reduced pressure, the obtained solid was collected, dried, and recrystallized from ethanol to give **14** as yellow crystals; m.p.: 57–59 °C, yield: 53%. IR (KBr, cm^−1^): 3240, 3200 (NH_2_), 2925, 2855 (CH_aliph._), 1591 (C=N or C=C), 1238 (C=S). ^1^H-NMR (400 MHz, DMSO-*d*_6_) δ (ppm): 8.51 (d, 1H, Ar-H, H_a_, *J* = 8.2 Hz), 7.82 (t, 1H, Ar-H, H_c_, *J* = 7.6 Hz, *J* = 7.8 Hz), 7.69 (d, 1H, Ar-H, H_d_, *J* = 7.6 Hz), 7.57 (t, 1H, Ar-H, H_b_, *J* = 7.6 Hz), 7.04 (s, 2H, NH_2_, exchangeable with D_2_O), 3.06 (t, 2H, N=CCH_2_, *J* = 7.2 Hz, *J* = 8.0 Hz), 1.81 (quintet, 2H, N=CCH_2_CH_2_, *J* = 7.6 Hz, *J* = 7.2 Hz), 1.42−1.32 (m, 4H, CH_3_CH_2_CH_2_), 0.88 (t, 3H, CH_3_, *J* = 6.8 Hz, *J* =7.2 Hz). ^13^C NMR (100 MHz, DMSO-*d*_6_) δ (ppm): 182.1, 155.5, 142.0, 134.5, 130.6, 128.1 (2), 127.4, 34.4, 31.3, 25.5, 22.4, 14.3. MS *m*/*z* (%): 247 (M^+^; 74.3). Anal. Calcd. for C_13_H_17_N_3_S (247.36): C, 63.12; H, 6.93; N, 16.99; S, 12.96. Found: C, 63.19; H, 6.96; N, 17.11; S, 12.82.

### 3.2. Cytotoxicity and Antiproliferative Evaluation

#### MTT Assay

The implement of MTT methodology for the antiproliferative screening of quinazolinone derivatives **5**–**14** along with compound **3** against two cell lines, namely, hepatocellular carcinoma (HepG2) and mammary gland (MCF-7) were obtained from ATCC through the Holding company for biological products and vaccines (VACSERA), Cairo, Egypt. The reference anticancer drug used was Doxorubicin. The MTT assay was carried out at the pharmacology department, Faculty of pharmacy, Mansoura University, Egypt according to the reported literatures [42,43,54]. The cells were cultured in a RPMI-1640 medium with 10% fetal bovine serum, followed by the addition of antibiotics (100 units/mL penicillin and 100 µg/mL streptomycin) at 37 °C in a 5% CO_2_ incubator. The cells were seeded in a 96-well plate at a density of (1.0 × 10^4^ cells/well) at 37 °C for 48 h under 5% CO_2_. Treatment of cells with different concentrations of compounds such as 100, 50, 25, 12.5, 6.25, 3.125, and 1.56 μM was carried out and placed in the incubator for 24 h. Then, 20 µL of MTT solution at 5 mg/mL was added and incubated for 4 h. DMSO (100 µL) was added into each well to dissolve the purple formazan formed. At 570 nm absorbance the colorimetric assay was measured and recorded by using a plate reader (BioTek EL ×800 Microplate Reader, BioTek Instruments, Inc, Winooski, VT, USA).

Calculation of the relative cell viability (%) = (A of treated samples /A of untreated sample) × 100.

### 3.3. Antioxidant Assay

#### 3.3.1. Antioxidant Activity Screening Assay

##### ABTS Method

By the bleaching of ABTS derived radical cations, the detections of antioxidant activities were estimated. The radical cation was prepared by the reaction of ABTS [2,2′-azino-bis(3-ethyl benzothiazoline-6-sulfonic acid)] (60 µL) with MnO_2_ (3 mL, 25 mg/mL) in a phosphate buffer solution (10 µM, pH 7, 5 mL). The solution was shaken for 3 min, centrifuged, filtered, and recorded at λ_max_ 734 nm the absorbance *A*_(control)_ of the resulting ABTS radical solution (green-blue). Upon the addition of the tested sample solution (20 µl) with different concentrations of compounds such as 200, 100, 50, 25, and 12.5 μM in spectroscopic grade MeOH/buffer (1:1 *v*/*v*) to the ABTS solution, the absorbance *A*_(test)_ was measured. The decreasing in the absorbance is expressed as % inhibition which was calculated according to the following equation [55]:

% Inhibition = [*A*_(control)_ − *A*_(test)_/*A*_(control)_] × 100 (1)

where; the reference and standard antioxidant compound in this test is the ascorbic acid solution (20 µL, 2 mM) and the blank sample was performed by the solvent without ABTS.

##### DPPH Method

According to the methodology described by Brand-Williams et al. [56], the measurement of the DPPH radical scavenging activity was implemented. The samples with different concentrations of compounds such as 200, 100, 50, 25, and 12.5 μM were allowed to react with the stable DPPH radical in ethanol solution. Whereas, the reaction mixture consisted of sample (0.5 mL), absolute ethanol (3 mL), and DPPH radical solution (0.3 mL) 0.5 mM in ethanol. DPPH is reduced when it reacts with an antioxidant compound, which can donate hydrogen. The changes in color (from deep violet to light yellow) were recorded [absorbance (*Abs*)] at λ_max_ 517 nm after 100 min of reaction using a UV-Vis spectrophotometer (Schimadzu Co., Tokyo, Japan). The blank solution was prepared by mixing ethanol (3.3 mL) and the sample (0.5 mL). Meanwhile, the mixture of ethanol (3.5 mL) and DPPH radical solution (0.3 mL) serve as a positive control.

The scavenging activity percentage (*AA* %) was determined according to Mensor et al. [57]:

*AA %* = 100 − [(*Abs*_(sample)_ − *Abs*_(blankl)_/*Abs*_(control)_) × 100] (2)

### 3.4. Computational Procedures

All theoretical calculations and results of the studied compounds were implemented by utilizing Gaussian(R) 09 D.01 [58] (Semichem Inc., Shawnee Mission, KS, USA) by applying the DFT operation with the hybrid functional B3LYP level [59,60] in conjunction with the 6−31G(d,p) basis set. The visualization of these results was achieved using GaussView 6.0.16 software (Semichem Inc., Shawnee Mission, KS, USA).

## 4. Conclusions

In conclusion, this work focused on the study of the antiproliferative and antioxidant activities in vitro in addition to the theoretical calculation of the DFT theory of some novel quinazolinone(thione) derivatives **6**–**14**. Two main points were the principal targets; firstly, by comparing the activities of quinazolinone and quinazolinthione derivatives. Secondly, comparing the activities of four series of Schiff bases, that have quinazolinone moiety. The results of this study imply that the quinazolinthione derivatives **6** and **14** have promising potent antiproliferative activity comparable with quinazolinone derivatives **5** and **9**, respectively. According to the DFT study, compounds **6** and **14** have a smaller energy gap and a higher chemical softness than that of compounds **5** and **9**, respectively. Additionally, screening of various aryl aldehyde hydrazone derivatives **(11a**–**d)** analogs exhibited that the potency increased with increasing the electron donating group in *p*-position due to increasing of the conjugated system, and that was supported by the DFT study. 

On the other hand, compounds **6** and **11d** showed promising antioxidant activity using ABTS assay. While in the DPPH assay, compounds **6**, **11d,** and **14** have showed potent activities comparable to the ascorbic acid which was used as a reference drug. Noteworthy, the results of both antiproliferative and antioxidant activities for each compound individually are nearly the same.

## Figures and Tables

**Table 1:** Cytotoxic efficacy of thirteen compounds against hepatocellular carcinoma (HePG2) and mammary gland (MCF-7) cell lines.

Compounds	In Vitro Cytotoxicity IC_50_ (µM)	
	HePG2	MCF-7
DOX	4.50 ± 0.3	4.17 ± 0.2
3	52.01 ± 3.3	47.53 ± 2.9
5	23.75 ± 1.9	21.98 ± 1.8
6	5.20 ± 0.5	6.88 ± 0.4
7	66.84 ± 4.0	54.23 ± 3.2
8	85.55 ± 4.5	70.86 ± 4.1
9	33.27 ± 2.5	28.69 ± 2.2
11a	41.92 ± 2.8	33.39 ± 2.5
11b	17.27 ± 1.4	19.68 ± 1.6
11c	12.54 ± 1.1	14.10 ± 1.3
11d	7.63 ± 0.6	8.60 ± 0.7
12	76.14 ± 4.3	59.15 ± 3.8
13	60.34 ± 3.8	56.28 ± 3.5
14	27.39 ± 2.3	10.78 ± 0.9

**Table 2:** Antioxidant activities of all synthesized compounds by using 2,2′-azino-bis(3-ethyl benzothiazoline-6-sulfonic acid (ABTS) and 2,2-diphenyl-1-picryl-hydrazyl-hydrate (DPPH) methods.

Compounds	ABTS IC_50_ (µM)	DPPH IC_50_ (µM)
Ascorbic acid	31.26 ± 0.20	16.83 ± 0.15
3	93.24 ± 0.70	52.14 ± 0.65
5	65.13 ± 0.46	41.16 ± 0.42
6	35.48 ± 0.23	19.25 ± 0.18
7	114.63 ± 0.73	59.43 ± 0.68
8	160.92 ± 0.94	92.36 ± 0.76
9	77.38 ± 0.56	46.23 ± 0.48
11a	89.10 ± 0.64	50.12 ± 0.54
11b	58.03 ± 0.41	38.47 ± 0.37
11c	52.87 ± 0.36	32.69 ± 0.29
11d	46.25 ± 0.28	22.91 ± 0.21
12	154.51 ± 0.85	73.03 ± 0.72
13	131.95 ± 0.77	65.58 ± 0.66
14	71.42 ± 0.52	26.87 ± 0.23

**Table 3:** Quantum chemical parameters of the selected compounds with Density Functional Theory (DFT) at B3LYP/6-31G (d,p) basis set.

	Comp. No.	5	6	9	14	11a	11b	11c	11d
	
*E* HOMO (eV)		−5.9432	−5.6352	−5.9405	−4.7724	−5.7277	−5.6363	−5.6717	−5.6602
*E* LUMO (eV)		−1.0917	−1.6515	−1.1298	−1.6836	−2.7301	−2.4983	−2.5709	−2.6009
(∆*E*) Energy gap (eV)		4.8515	3.9837	4.8107	3.0888	2.9976	3.1380	3.1008	3.0593
(*I*) Ionization energy (eV)		5.9432	5.6352	5.9405	4.7724	5.7277	5.6363	5.6717	5.6602
(A) Electron affinity (eV)		1.0917	1.6515	1.1298	1.6836	2.7301	2.4983	2.5709	2.6009
(*ƞ*) Chemical hardness (eV)		2.4258	1.9919	2.4054	1.5444	1.49881	1.56901	1.5504	1.52969
(*S*) Chemical softness (eV^−1^)		0.4122	0.5020	0.4157	0.6475	0.6672	0.6374	0.6450	0.6537
(*E*_T_) Binding energy (a.u)		−689.79	−1012.73	−745.11	−1068.05	−1473.81	−1128.73	−1203.94	−1318.44
(µ) Dipole moment (D)		3.2867	3.4641	1.764	1.852	3.2722	4.4472	4.1944	6.232
